# Clinical and molecular features of NDM-producing *Acinetobacter baumannii* in a multicenter study in Israel

**DOI:** 10.1186/s12941-023-00607-w

**Published:** 2023-06-30

**Authors:** Amos Adler, Hiren Ghosh, Andrea Gross, Amit Rechavi, Michal Lasnoy, Marc V. Assous, Yuval Geffen, Basel Darawsha, Yonit Wiener-Well, Anat Alony, Hajo Grundmann, Sandra Reuter

**Affiliations:** 1 Clinical Microbiology, Tel Aviv Sourasky Medical Center, Tel Aviv, Israel; 2 Department of Epidemiology and Preventive Medicine, School of Public Health, Faculty of Medicine, Tel Aviv University, Tel Aviv, Israel; 3 Institute for Infection Prevention and Hospital Epidemiology, Medical Center - University of Freiburg, Freiburg, Germany; 4 Ruppin Academic Center, Ruppin, Israel; 5 Laboratory of Clinical Microbiology, Faculty of Medicine, Shaare Zedek Medical Center, Hebrew University of Jerusalem, Jerusalem, Israel; 6 Rambam Medical Center - Haifa, Haifa, Israel; 7 Infectious disease unit, Sha’are Zedek Medical Center, Faculty of Medicine, Hebrew University of Jerusalem, Jerusalem, Israel

**Keywords:** *Acinetobacter baumannii*, NDM, Carbapenem, Whole-genome sequencing, Transmission

## Abstract

**Background:**

NDM-producing *Acinetobacter baumannii* (NDMAb) were reported sporadically worldwide but little is known about the transmission, epidemiology and clinical features of NDMAb-infected patients. The goals of this study were to characterize (1) the epidemiology and clinical features of NDMAb–infected patients; (2) the microbiological and molecular features of NDMAb isolates and (3) the transmission networks of NDMAb within healthcare facilities.

**Methods:**

The study was conducted at the Tel-Aviv Sourasky, Rambam and Sha’are-Zedek Medical centers (TASMC, RMC and SZMC, respectively) in Israel. All cases detected between January 2018 and July 2019 were included. Phylogenetic analysis was based on core genome SNP distances. Clonal transmission was defined according to molecular (≤ 5 SNP) and epidemiological criteria (overlapping hospital stay). NDMAb cases were compared at a ratio of 1:2 with non-NDM carbapenem-resistant *A. baumannii* (CRAb) cases.

**Results:**

The study included 54 NDMAb-positive out of 857 CRAb patients, including 6/179 (3.3%) in TASMC, 18/441 (4.0%) in SZMC and 30/237 (12.6%) in RMC. Patients infected by NDMAb had similar clinical features and risk factors as patients with non-NDM CRAb. The length-of-stay was higher in NDMAb cases (48.5 days vs. 36 days, respectively, p = 0.097) and the in-hospital mortality was similarly high in both groups. Most isolates (41/54, 76%) were first detected from surveillance culture. The majority of isolates harbored the *bla*_NDM−2 gene_ allele (n = 33), followed by the *bla*_NDM−1_ (n = 20) allele and the *bla*_NDM−4_ allele (n = 1). The majority of isolates were related within the ST level to other isolates in SZMC and RMC: 17/18 and 27/30 isolates, respectfully. The common ST’s were the *bla*_NDM−1_ harboring ST-2 (n = 3) and ST-107 (n = 8) in SZMC and the *bla*_NDM−2_ harboring ST-103 in SZMC (n = 6) and in RMC (n = 27). All *bla*_NDM_ alleles were located within a conserved mobile genetic environment flanked by the IS*Ab125* and IS91 family transposon. Clonal transmission was identified in most hospital-acquired cases in RMC and SZMC.

**Conclusion:**

NDMAb constitutes a minor part of CRAb cases and are clinically similar to non-NDM CRAb. Transmission of NDMAb occurs mostly by clonal spread.

**Supplementary Information:**

The online version contains supplementary material available at 10.1186/s12941-023-00607-w.

## Introduction

Carbapenem-resistant *Acinetobacter baumannii* (CRAb) constitute a major threat to public health and are considered as one of the top priority pathogens [1, 2]. CRAb typically cause infections in patients who have been cared for in healthcare settings, especially in those who require invasive medical devices in intensive care units [3]. In Israel, CRAb continue to be a major cause of morbidity in healthcare facilities [4, 5], including blood stream infections [6].

Carbapenem resistance in *A. baumannii* is typically mediated by the OXA-type carbapenemase, with *bla*_OXA−23_ being the most common [7]. In addition to carbapenem resistance, CRAb isolates are extensively resistant to multiple antimicrobial agents [8], and thus have only a few therapeutic options, such as polymyxins. In addition to OXA-type carbapenemases, metallo-β-lactamases, including IMP, VIM and NDM, have also been found in CRAb [8].

Sporadic cases of NDM-producing *A. baumannii* (NDMAb) have been reported from several countries, including the Indian subcontinent [9], China [10], the Middle East [11, 12], Europe [13] and the USA [14]. NDMAb was initially found in Israel as early as 2009 [15] in surveillance cultures that were collected from elderly patients in rehabilitation centers. Since then, a small survey has identified NDMAb in 5.1% of CRAb isolates in one center. Despite the global spread of NDMAb, little is known about the epidemiology and clinical features of NDMAb-infected patients, as well as the molecular epidemiology and transmission networks of NDMAb in healthcare facilities.

The goals of this study were to characterize in a multicenter study (1) the epidemiology and clinical features of NDMAb – infected patients, compared with non-NDM CRAb infected patients; (2) the microbiological and molecular features of NDMAb isolates and (3) the transmission networks of NDMAb within healthcare facilities.

## Methods

### Setup and settings

The study was conducted at three university-affiliated tertiary care centers in the largest cities in Israel: (1) Tel Aviv Sourasky Medical Center (TASMC), a 1400-bed, center in Tel Aviv; (2) Rambam Medical Center (RMC), a 1000-bed center in Haifa and (3) the Sha’are Zedek Medical Center (SZMC), a 1000-bed center in Jerusalem. There are no official guidelines for CRAb screening in Israel, but for the most part, patient screening policies were similar to those of CPE and followed the guidance of the Israeli Ministry of Health [16]. Briefly, surveillance cultures were collected by either rectal or pharyngeal swabs in patients following either: (i) patients admitted after recent hospital stay in another institution (admission screening); (ii) recent contact with another confirmed CRAb infected patient (contact screening); and (iii) repetitive, high-density screening in specific high-risk wards, e.g. intensive care unit.

### Patients and data collection

All isolates (first isolates of every species) of CRAb from surveillance or clinical diagnostic cultures of hospitalized patients detected between January 2018 and July 2019 were included and transferred periodically to the TASMC laboratory for identification of NDMAb (see below). Due to unexpected refrigeration error, not all TASMC CRAb isolates were preserved and therefore transmission analysis was not performed in TASMC. Data was collected retrospectively for (1) all NDMAb patients (study group) and (2) non-NDM CRAb cases (control group), chosen at a 1:2 ratio. The control group were matched with the study group for (1) medical center, (2) time period (within six months); (3) gender and (4) age (+/- 5 years).

Data were collected from the patients’ electronic records: (1) microbiological information, including all sites and isolation dates; (2) admission and discharge dates of individual patient’s hospital episodes; (3) demographic data; (4) comorbidities, including the Charlson Comorbidities Score (CCS); (5) previous exposure to health care facilities and treatments and (6) outcome measures, including subsequent clinical infection, hospital length of stay and in-hospital mortality.

### Microbiological methods and detection of carbapenemases

Swabs from surveillance screening were inoculated on selective, differential media (TASMC and RMC: CHROMAgar™ mSuperCARBA™ agar; SZMC: CHROMAgar™ KPC). Suspicious colonies grown on the medium (i.e., non-chromogenic, oxidase-negative colonies) were identified and antimicrobial susceptibility testing (AST) to meropenem was carried out. Clinical culture was performed in accordance with the American Society of Microbiology guidelines [17]. Species determination was performed using a VITEK® MS matrix-assisted laser desorption/ionisation time-of-flight (MALDI-TOF) system (bioMérieux, Marcy-l’Étoile, France). PCR for the *bla*_NDM_ gene was done in TASMC on all CRAb isolates [18]. AST was done in TASMC for all NDMAb isolates from the three centers using the VITEK®2 system and the Etest® gradient method (for imipenem and meropenem only) (bioMérieux, Marcy-l’Étoile, France) and was interpreted according to Clinical and Laboratory Standards Institute (CLSI) criteria [19].

### Next generation sequencing, phylogenetic analyses and identification of potential genomic clusters

DNA was extracted using the High Pure PCR Template Preparation Kit from Roche. Sequencing was performed using an Illumina MiSeq with 2 × 150 bp paired end sequencing after Nextera DNA Prep library preparation. Taxonomic assignment check was done with Kraken v0.10.5-beta and the minikraken 4GB database [20]. Isolates were mapped to reference genome A1 (CP010781) using smalt version 0.7.6 (https://www.sanger.ac.uk/science/tools/smalt-0). Sequence reads were assembled using SPAdes v3.11.1 (http://cab.spbu.ru/software/spades/), with k-mer sizes 21, 33, 55, 77, 99, 109, and 123. Assemblies were then filtered to only include contigs with a minimum of 500 bp. Multi-locus sequence typing was carried out with mlst v2.10 (https://github.com/tseemann/mlst) using the respective typing schemes where applicable, and new ST were assigned. QC criteria were allocation of reads to the expected species using Kraken and the minikraken 4GB reference database [20], coverage > 30X, appropriate genome size, number of contigs < 500, largest contig > 100,000 bp, N50 > 100,000 bp, and identification of MLST alleles.

For phylogenetic reconstructions, single-nucleotide polymorphisms (SNPs) were filtered from the mapping data with GATK (https://gatk.broadinstitute.org/hc/en-us), and only SNPs with at least 4 reads coverage and present in > 75% of reads were included. These variant filtered files were then converted to a fasta file, where SNP sites and absent sites (N) were replaced compared to the reference genome. All isolates were then combined to an alignment, and regions associated with mobile genetic elements were removed (i.e. capsule locus, phages, Tn/IS elements; https://github.com/andrewjpage/remove_blocks_from_aln). SNP sites were extracted and the resulting alignment used to reconstruct a maximum likelihood phylogeny with RAxML v8.2.4. Trees were visualised using iTOL.

Sequencing statistics and accession numbers can be found in Table S1; the average sequencing coverage was 59X, and the assembly size 4.1 Mb in 72 contigs.

To determine potential transmission events based on the genome data, we visually inspected and identified closely related groups of isolates within the same ST. We analysed each of these groups by choosing the isolates with the best assembly as a new reference to map against, and visually checked the identified SNPs for mapping artefacts or recombination. We defined recent ancestral relatedness and hence contemporary transmission events (direct transmission) for isolates with 5 or less SNPs between them.

For Nanopore sequencing of the selected samples, the sequencing library was prepared, using a Native barcoding amplicons (SQK-LSK109) kit (Oxford Nanopore Technologies Ltd., UK) and the sequencing were performed using a Gridion sequencer and a FLO-MIN106 Flow Cell version R9.4.1 (Oxford Nanopore Technologies Ltd., UK). The raw data were demultiplexed with Guppy software (v6.0.7) The quality of the demultiplexed reads was analyzed with minion_qc (https://github.com/roblanf/minion_qc) and reads longer than 1000 bp were filtered out using Filtlong (v0.2.1). The high quality reads were assembled using Flye (v2.9). The assembly was polished using two rounds of Medaka (v. 1.4.4) using default settings.

### Analysis of NDM genes and their associated mobile genetic elements

Antibiotic resistance genes and plasmid Inc types were identified using Abricate (version 0.9.8, https://github.com/tseemann/abricate) against the database Resfinder [21] and Plasmidfinder [22] with the default parameter settings. We focused our analysis on the plasmid diversity of *bla*_NDM−1_ encoding isolates. The contig encoding the *bla*_NDM_ gene was extracted and annotated using Prokka (version1.14.5) [23] and manually curated using blastp. A pan genome matrix was generated by Roary (v.3.13.0) [24] with default parameter settings. The gene dissimilarity matrix output was clustered using the hclust package in R (version 4.0.2). The genetic environment surrounding the *bla*_NDM_ gene was examined using Clinker (version 0.0.23) [25] in order to determine the uniformity of the NDM context. Initially all modules were aligned independently, and the most conserved genomic structure of each module was determined in order to compare the differences between the modules. We then also used the curated ONT assembly to compare the blaNDM genetic environment with 5 genes upstream and 5 genes downstream.

### Identification of potential transmission events

Hospital episodes (date of admission until date of discharge) and patient locations (i.e., wards) were reconstructed by a contact network to identify temporal and spatial proximity between patients. Patients were categorized as colonized/infected pre admission vs. potentially post-admission based on the results of admission screens (up to two days from admission). Contact networks for each hospital were filtered for potential transmission events between patients carrying or infected with isogenic isolates (core genome distance ≤ 5 SNP) and same ward overlapping treatment episodes.

## Results

### Microbiological features of NDMAb isolates

During the study, 857 patient-unique CRAB isolates were collected and screened by *bla*_NDM−1_-PCR. We identified 54 NDMAb cases, including 6/179 (3.3%) in TASMC, 18/441 (4.0%) in SZMC and 30/237 (12.6%) in RMC. Except for one *A. pittii* isolate from TASMC, all other isolates were *A. baumannii* senu strictu. Most isolates (41/54, 76%) were first detected from surveillance culture, mostly from rectal swabs (n = 40; Table 1). In addition to *bla*_NDM_, six *bla*_OXA−23_ and ten *bla*_OXA−58_ were detected. All the isolates (except one) also harbor the *bla*_ADC−25_ cephalosporinase gene.

**Table 1:** Demographics and comorbidity factors of patients infected with NDM-producing vs. non-NDM Carbapenem-resistant *Acinetobacter baumannii*

Variable	NDM-*A. baumannii* (n = 54)	Non-NDM *A. baumannii* (n = 108)	p-value
Female gender, N (%)	22 (41)	44 (41)	1.0
Age (years), mean (S.D.)	67.5 (19.6)	66.4 (19.5)	0.73
Hospital, N (%) SZMC RMC TASMC	18 (33.3) 30 (55.6) 6 (11.1)	36 (33.3) 60 (55.6) 12 (11.1)	1.0
LTCF residency, N (%)	10 (18.5)	34 (31.5)	0.08
Charlson Clinical Score (SD)	3.81 (2.91)	3.46 (2.76)	0.45
Recent hospitalization (3 months), N (%)	31 (57)	64 (59)	0.82
Recent surgery (3 months), N (%)	12 (22.2)	34 (31.5)	0.21
Recent chemotherapy (1 month), N (%)	4 (7.4)	10 (9.3)	0.69
Recent antimicrobial use (1 month), N (%)	51 (94)	89 (82)	0.035
Recent use of urinary catheter (1 week), N (%)	33 (61)	61 (56)	0.57
Recent use of CVC (1 month), N (%)	21 (39)	37 (34)	0.56
Recent mechanical ventilation (1 week), N (%)	21 (39)	48 (44.4)	0.5
First isolation from surveillance, N (%)	41 (76)	78 (72)	0.61

All NDMAb isolates were resistant to ceftazidime, piperacillin-tazobactam, imipenem and meropenem; 4 isolates (7%) were susceptible to ampicillin-sulbactam and all isolates were susceptible to colistin (MIC ≤ 2 mg/L) (according to EUCAST criteria, [26]). The numbers of isolates that were susceptible to other agents were: gentamicin- 3 (5%), tobramycin-12 (21%), ciprofloxacin and levofloxacin- 3 (5%), minocycline- 55 (100%), tigecycline- 51 (92%) and trimethoprim-sulfamethoxazole- 3 (5%) (Table S2).

### Clinical and epidemiological features of NDMAb cases

The demographic and clinical characteristics and the outcome of these cases is presented in Tables 1 and 2, respectively. Among the NDMAb cases, post-admission acquisition were determined in 3/6 (50%), 13/18 (72%) and 24/30 (80%) in TASMC. SZMC and RMC, respectively.

**Table 2:** Clinical features and outcome of patients infected with NDM-producing vs. non-NDM Carbapenem-resistant *Acinetobacter baumannii*

Variable	NDM-*A. baumannii* (n = 54)	Non-NDM *A. baumannii* (n = 108)	p-value
Any isolation from clinical site, N (%)	19 (35)	45 (41)	0.42
Any isolation form blood, N (%)	5 (9.3)	8 (7.4)	0.68
Isolation from clinical sites in carriers, N (%)	6 (14.6)	16 (20.5)	0.43
Time from colonization to clinical infection, median days (95% C.I.)	13 (2.2–23.8)	7 (3-10.9)	0.31
In-hospital mortality, N (%)	23 (42.6)	36 (33)	0.24
Length of stay, mean (S.D.)	48.5 (59)	36 (36.5)	0.097

NDMAb patients had similar risk factors overall in comparison with non-NDM CRAb patients, with minor exceptions. Patients in both groups had significant underlying diseases and various exposures to healthcare facilities. Non-NDM CRAb patients had lower rate of recent use of antimicrobials (94% vs. 82%, respectively, p = 0.035) (Table 1) and a higher rate of prior LTCF residence compare with NDMAb patients (31.5% vs. 18.5.5%, respectively, p = 0.08) although this difference was not statistically significant. Most patients were admitted due to illnesses not related to CRAb infection (data not shown) and indeed, in the majority of patients CRAb was first detected in surveillance culture.

Similar rates of patients in both groups had CRAb isolated at various clinical sites (35% and 41% in NDMAb and non-NDM CRAb, respectively) including the isolation in blood cultures that was documented in 9.3% and 7.4% (in NDMAb and non-NDM CRAb, respectively). With the exception of blood isolation, it was extremely problematic to determine the clinical relevance of the isolation in other clinical sites. Among colonized patients, subsequent clinical cultures were detected in higher rates and after shorter duration in non-NDM CRAb patients, but the difference was not statistically significant. The LOS was higher in NDMAb cases (48.5 days vs. 36 days, respectively, p = 0.097) and the in-hospital mortality was similarly high in both groups, likely representing the overall high rate of co-morbidities.

### ***bla***_NDM_***allele and flanking mobile genetic elements***

**Fig. 1:**
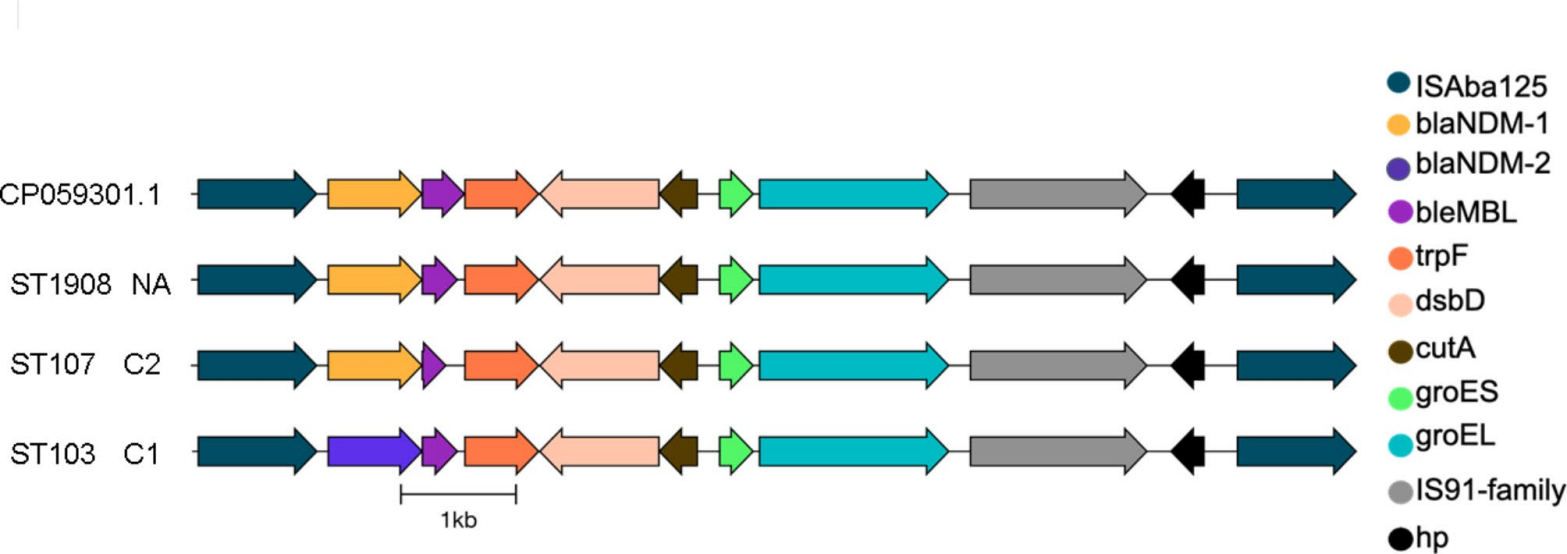
Schematic representation of the *bla*_NDM_ genetic environment. Module-specific representative isolates from MGE module C1 and C2 are presented, in comparison to isolate CP059301.1. Conserved genes were indicated by similar color shading

**Fig. 2:**
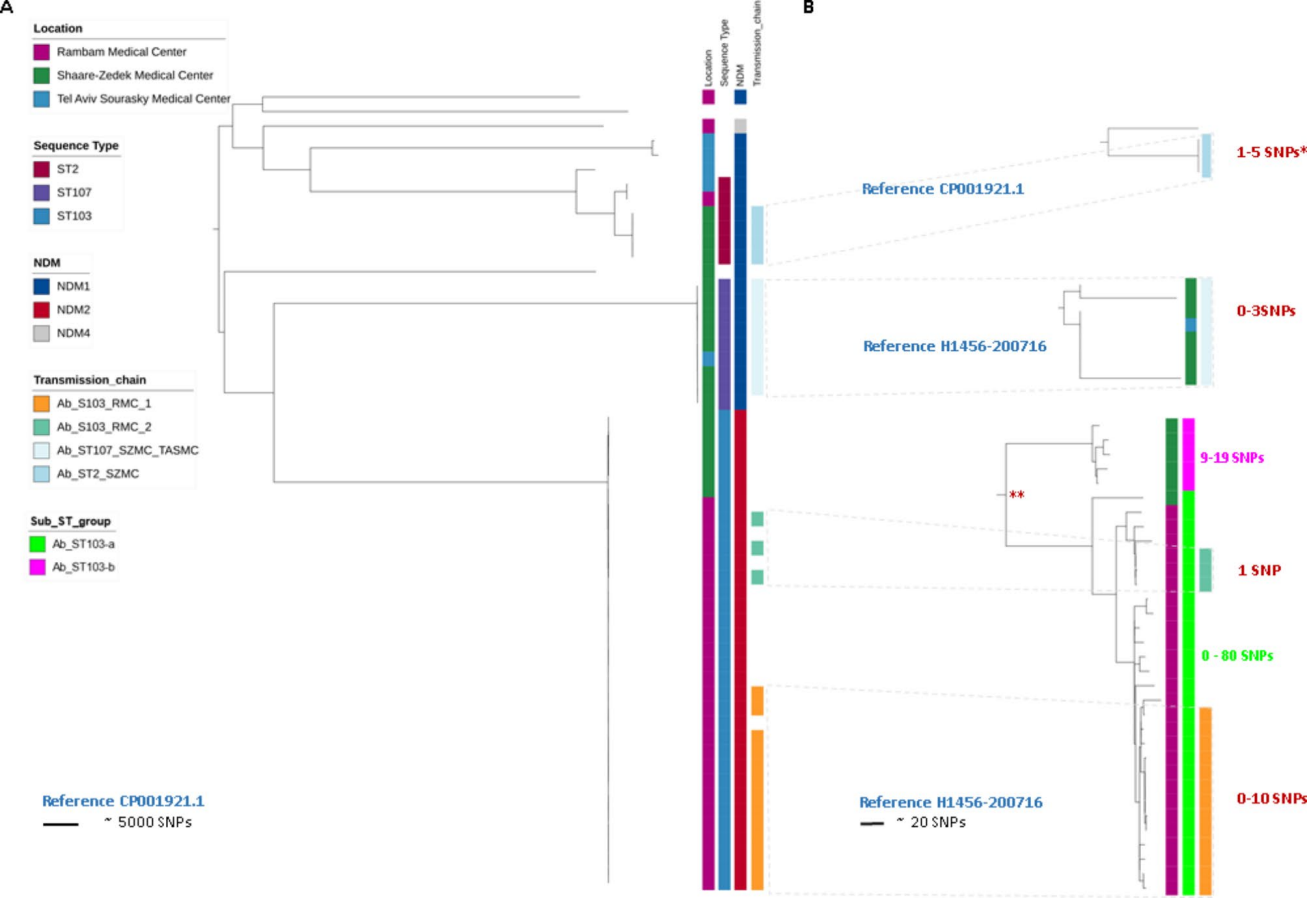
Phylogenetic reconstruction of NDM-producing *A. baumannii.* (A) Overview phylogeny of all sequenced isolates. Distance is displayed in terms of differences in the number of SNPs. Location, NDM type and sequence type is displayed as well as affiliation to sub-ST-groups and transmission chains. (B) Detailed view of proposed transmission clusters based on cluster-specific reference-mapping. *difference to other ST2 isolates > 1,0.000 SNPs; **minimum difference between branches = 121 SNPs

Three alleles of *bla*_NDM_ were identified, corresponding to specific ST (Table 3 and S1, Figs. 1 and 2), *bla*_NDM−1_ (n = 20), *bla*_NDM−2_ (n = 33) and *bla*_NDM−4_ (n = 1). *bla*_NDM−2_ was associated with ST103, *bla*_NDM−4_ with ST1017, and *bla*_NDM−1_ with various sequence types. The *bla*_NDM_ genes of all three alleles were located within a conserved mobile genetic element (Fig. 1) that contained two IS*Ab125* elements with one end flanked by the IS*91* family elements that was located on the bacterial chromosome. Due to the short-read nature of our data it is not possible to determine whether the insertion site is the same in all cases. This structure was absent in a three-isolate cluster in SZMC, Ab_ST2_SZMC that included only half of the structure.

**Table 3:** Distribution of NDMAb isolates according to center, *bla*_NDM_ allele, sequence type, and clonal cluster

Center (n)	*bla*_NDM_ allele (n)	ST^1^ (n)	Cluster (n)
TASMC (6)	1 (6)	85 (2), 2, 396^2^, 570	None
		107	ST107_SZMC_TASMC (1)^3^
SZMC (18)	1 (12)	2 (3)	ST2_SZMC (3)
		107 (8)	ST107_SZMC_TASMC (7)^3^
		1908	None
	2 (6)	103 (6)	None
RMC (30)	1 (2)	2, 1907	None
	2 (27)	103 (27)	ST103_RMC_1 (13), ST103_RMC_2 (3)
	4 (1)	1017	None

^1^- ST- sequence type; ^2^-*Acinetobacter pittii*;^3^-cluster includes patients from two hospitals

### Clonal structure and transmission on NDMAb

The dissemination of NDMAb was mostly clonal, as the majority of isolates were related within the ST level to other isolates in SZMC and RMC (Table 3; Fig. 2): 17/18 and 27/30 isolates, respectfully. The common ST’s were the *bla*_NDM−1_ harboring ST-2 (n = 3) and ST-107 (n = 8) in SZMC and the *bla*_NDM−2_ harboring ST-103 in SZMC (n = 6) and in RMC (n = 27).

Within the same ST’s, 10/17 and 16/27 isolates were clustered as presumptive transmission chains by WGS in SZMC and RMC, respectfully (Table 2). Of these, 9/10 and 13/16 of the cases were acquired post-admission in SZMC and RMC, respectfully. In SZMC, 9 of the cases that were acquired post-admission could be traced to another patient, having parallel hospitalization time and location (n = 6) or by time only (n = 3). In RMC, all of the ST103_RMC_2 cluster cases (n = 3) were acquired post-admission and could be traced to another patient, having parallel hospitalization time and location (n = 2) or by time only (n = 1). Of the ST103_RMC_1 cluster (n = 13), ten cases were acquired post-admission. Of these ten cases, seven could be traced to another patient, having parallel hospitalization time and location (n = 5) or by time only (n = 2). Of note, all of the 13 ST103_RMC_1 cluster cases were hospitalized at some point of the study in one Internal Medicine ward. Hence, it is likely that all of these cases were acquired in the same ward and were only detected later. Moreover, both RMC ST103 clusters are related between 20 and 50 SNPs. This would not be indicative of recent within-hospital transmission but would suggest a locally-circulating clone.

In TASMC, no spreading clones were identified by WGS or epidemiology, probably due to the small number of cases and incomplete collection (see ‘methods’). Interestingly, one case was clonally-related (0 SNPs to some cluster members) to the dominant clone in SZMC (ST107_SZMC_TASMC).

## Discussion

In this multicenter study, we aimed to fill important gaps in the knowledge regarding the epidemiology, clinical features and the transmission modes of NDMAb in Israel. Our multicenter, 18-month survey was able to provide novel and extensive data pertaining these questions.

NDMAb was found to constitute a minor share of the CRAb microbial population, i.e., between 3.3 and 12.6%. This relatively low ratio is similar to our previous findings [27] of 5.1% and to previous studies from Palestine [28] and Lebanon [29]. In Egypt, a report from 2015 found NDMAb among 39.1% of all CRAb [30], indicating it’s dissemination potential. This report [30] also found very high rate of co-occurrence of NDM with OXA-23 in these isolates (53/59, 89%), which was identified in only 6/54 (11%) of NDMAb isolates in our study. Co-occurrence with OXA-58 was found in ten isolates (18%), similar to previous report from China [31].

Patients infected or colonized by NDMAb were for the most part indistinguishable from patients with non-NDM CRAb, when matched by age and gender. Both groups had high rate of co-morbidities, manifested by the high mean CCS and high rate of previous exposure to various healthcare related procedures, similar to previous reports from Israel [4]. Some minor differences existed, such as lower rate of LTCF residence in the NDMAb group, but this might be due to epidemiological differences in the local LTCF. Since in most cases NDMAb was first detected by surveillance culture, it is not surprising that the admission causes were mostly unrelated and thus the high in-hospital mortality was likely a reflection of previous underlying conditions. In both types of CRAb, a subsequent isolation from clinical site was uncommon as was bacteremia that appeared in less than 10% of cases. This rate of subsequent clinical infection in carriers is similar to a previous study from Israel [4] and much lower compared with the rate of 108/200 (54%) subsequent bacteremia cases in CRAb carriers reported from Korea [32] or the 69% rate of clinical infection in carriers reported from the USA [33]. These differences between the countries are extensive and can be explained by differences in CRAb surveillance policies: a more restricted policy aimed for high-risk patients, may detect patients that are more prone to develop clinical infection.

The molecular analysis revealed that the NDMAb population was mostly clonal, with almost all isolates belonging to one of the main ST in their respective institutions. This clonal structure is in a stark contrast with the highly heterogeneous population structure of NDM-producing Enterobacterales that we found in the same institutions [34]. The dominant ST found in our study, ST 2,, ST 85, ST 107 and especially ST 103, were already reported from Israel [15] as well as from other Middle Eastern [12, 29, 35, 36] countries. Interestingly, OXA-58 producing ST 103 was identified as a “successful” clone in France [37], whereas the dominant international clones IC1 and IC2 are underrepresented in NDMAb. Together, this data suggests that several NDMAb clones have disseminated throughout the Middle East as early as 2009 and remained dominant to this day. However, since their rate compared with non-NDM CRAb remains relatively low, it seems that other clones predominate the overall CRAb microbial population.

Within the three major STs, we identified four putative transmission clusters. Almost all of them could also be traced to another patient. This goes in concordance with the clonal nature of NDMAb spread already discussed. The few cases that could not be linked epidemiologically might be related to “missing links” of NDMAb carriers. Of note, due to the long standing presence of some of these clones in Israel [15], it is possible that some of the transmission had occurred in LTCF.

All three *bla*_NDM_ alleles in our study showed the same MGE structure, with a similar gene composition downstream of the *bla*_NDM_ gene as described in previous reports [38]. These MGE included the IS*Aba125* as was featured in many previous reports [15, 38, 39] and were located within the bacterial chromosome. This fact gives further support to our hypothesis of clonal, rather than MGE-related dissemination of NDMAb. IS*91* elements were found in *A. baumannii* in association with various resistance genes [40, 41], but not with the *bla*_NDM_ gene Interestingly, we identified an almost identical plasmid-borne MGE structure in *A. baumannii* genome CP059301.1.

There are several limitations regarding the ability of our study to answer some of its goals. First, the relatively small number of patients might have precluded the identification of risk factors, e.g. prior LTCF residence. However, the general features of these patients seemed indistinguishable compared with non-NDM CRAb patients and these risk factors were therefore likely to be related to different epidemiological circumstances rather than actual biological differences. Second, the transmission analysis might have been incomplete due to potential “missing links” of transmission (e.g., unidentified carriers). Still, our data is sufficient to substantiate clonal spread as the main mode of transmission of NDMAb.

In conclusion, our study answers the questions that we aimed to investigate: the epidemiology and clinical features of NDMAb-infected patients are almost indistinguishable to those with non- NDM CRAb; the transmission of NDMAb had occurred via clonal expansion, both on the hospital level and most likely also on a national level.

## Electronic supplementary material

Below is the link to the electronic supplementary material.

Supplementary Material 1

Supplementary Material 2

## Data Availability

The NGS datasets generated during the current study are available in the European Nucleotide Archive project number PRJEB58043.
