# Supplementary material for: Clinical and molecular features of NDM-producing Acinetobacter baumannii in a multicenter study in Israel
Source: Ann Clin Microbiol Antimicrob. 2023 Jun 30;22:52. doi: 10.1186/s12941-023-00607-w (PMC10314562; doi:10.1186/s12941-023-00607-w)
Supplement: Supplementary file 2 — Supplementary Material 2 [file 12941_2023_607_MOESM2_ESM.docx]

**Table S2.** **Antimicrobial susceptibility testing results of 54 NDM-producing *Acinetobacter baumannii* isolates.**

| Antimicrobial agent | MIC values (mg/L), median | MIC values (mg/L), range | Susceptible isolates, n (%) |
| --- | --- | --- | --- |
| Ampicillin-sulbactam | 16 | 8-≥32 | 4 (7) |
| Ceftazidime | ≥64 | ≥64 | 0 |
| Ciprofloxacin | ≥4 | ≤0.25-≥4 | 3 (5) |
| Colistin | ≤0.5 | ≤0.5 | 54 (100)^1^ |
| Gentamicin | ≥16 | ≤1-≥16 | 3 (5) |
| Imipenem | 8 | 4-≥16 | 0 |
| Levofloxacin | ≥8 | ≤0.25-≥8 | 3 (5) |
| Meropenem | 8 | 4-≥16 | 0 |
| Minocycline | 2 | ≤1-4 | 54 (100) |
| Piperacillin-tazobactam | ≥128 | ≥128 | 0 |
| Trimethoprim-sulfamethoxazole | ≥320 | ≤20-≥320 | 3 (5) |
| Tigecycline | 2 | ≤0.5-4 | NA^2^ |
| Tobramycin | 8 | ≤1-≥16 | 12 (22) |

^1^-according to EUCAST criteria; ^2^-NA- no CLSI breakpoints are available for tigecycline.
